# Adopting Sustainable *Jatropha* Oil Bio-Based Polymer Membranes as Alternatives for Environmental Remediation

**DOI:** 10.3390/polym14163325

**Published:** 2022-08-16

**Authors:** Nur Haninah Harun, Zurina Zainal Abidin, Umar Adam Majid, Mohamad Rezi Abdul Hamid, Abdul Halim Abdullah, Rizafizah Othaman, Mohd Yusof Harun

**Affiliations:** 1 Department of Chemical and Environmental Engineering, Faculty of Engineering, Universiti Putra Malaysia, Serdang 43400, Malaysia; 2 Department of Chemistry, Faculty of Science, Universiti Putra Malaysia, Serdang 43400, Malaysia; 3 Department of Chemical Science and Food Technology, Universiti Kebangsaan Malaysia, Bangi 43600, Malaysia

**Keywords:** membrane, filtration, renewable, biodegradable, sustainable, *Jatropha*, copper, response surface methodology

## Abstract

This study aimed to optimize the removal of Cu(II) ions from an aqueous solution using a *Jatropha* oil bio-based membrane blended with 0.50 wt% graphene oxide (JPU/GO 0.50 wt%) using a central composite model (CCD) design using response surface methodology. The input factors were the feed concentration (60–140) ppm, pressure (1.5–2.5) bar, and solution pH value (3–5). An optimum Cu(II) ions removal of 87% was predicted at 116 ppm feed concentration, 1.5 bar pressure, and pH 3.7, while the validated experimental result recorded 80% Cu(II) ions removal, with 95% of prediction intervals. A statistically non-significant term was removed from the analysis by the backward elimination method to improve the model’s accuracy. Using the reduction method, the predicted R^2^ value was increased from −0.16 (−16%) to 0.88 (88%), suggesting that the reduced model had a good predictive ability. The quadratic regression model was significant (R^2^ = 0.98) for the optimization prediction. Therefore, the results from the reduction model implied acceptable membrane performance, offering a better process optimization for Cu(II) ions removal.

## 1. Introduction

To date, the demand for fresh water is increasing due to the rapid growth of the human population and the booming of industrial sectors [1]. The release of harmful waste, mainly from industrial activities such as coal mining or metal processing, in the form of water-soluble toxic metal waste, can upset water quality [2]. Several studies have also reported the discharge of acidic wastewater, with pH values ranging from 1.8 to 4.9, originating from industrial facilities associated with metal-based mines and metal-plating processes [3,4,5,6]. This toxic effluent, unfortunately, can easily leach into the earth’s surface and groundwater, potentially harming not only the environment, but also living organisms [7]. For example, waste rock containing sulfur-bearing minerals undergoes chemical reactions with surface water, creating an acidic environment to promote heavy metals leaching [6,8]. Similarly, heavy metal sludges from the metal-plating process, which are commonly disposed of in landfills, can also leak into the disposal soil area [7,9,10]. In other cases, stormwater flowing through contaminated area may cause a non-point source of pollution that delivers sediments, toxic contaminants, and the leaching of heavy metals [11,12,13,14]. It is important to note that many heavy metals are water-soluble, possess high toxicity, and are carcinogenic. Non-biodegradable heavy metals beyond an acceptable limit in water sources, if consumed, can lead to organ damage, cancer, nervous system damage, and in some extreme cases, even lead to death [15,16].

Various methods for metal ion removal have been applied, including flocculation, membrane filtration, photocatalysis, and coagulation [17,18,19]. Among these technologies, membrane filtration is favored due to its small footprint and chemical usage, with minimal impact on the environment compared to the conventional method that generates a significant amount of sludge after process treatment [19]. Moreover, due to its versatility, a variety of membrane processes have been developed to suit various types of wastewater streams [20]. A membrane consists of a thin layer supported on a porous matrix that acts as a selective barrier to prevent the passage of larger molecules. Selectivity of membranes is governed by many parameters, but is not limited to the rate of molecular diffusion through the membrane layer and interaction between the molecules with the membrane surface (molecular adsorption) [21,22].

Generally speaking, a synthetic membrane can be categorized based on its material type, namely inorganic or organic membranes [23]. The former is fabricated from inorganic materials, such as ceramics, zeolites, palladium alloys, metals, and oxides [24,25]. Inorganic membranes are often fabricated on microporous supports to provide high mechanical stability. The latter, on the other hand, are usually prepared from polymer-based materials, such as polyvinylidene difluoride, polyamide, polyimide, polysulfone, and polypropylene [26]. Despite being inexpensive and having superior thermal and chemical stabilities, inorganic membranes are not as prevalent in the commercial membrane market for wastewater treatment as compared to polymeric membranes (organic), due to their difficulty in forming high surface-to-volume modules. Polymeric membranes are extensively employed in wastewater treatment (research or commercial use) due to the flexibility in selecting membrane structure and configuration (spiral wound, hollow fiber, etc.), ease of processing, material diversity, and low cost of manufacturing [27]. It is worth mentioning that synthetic organic membranes can also be processed from synthetic monomers or chemically modified natural polymers, such as polylactic acid (PLA), cellulose, chitosan, starch [28,29,30], and vegetable oil [31,32], which are eco-friendly, non-toxic, and sustainable [33,34]. With diverse chemical properties, organic polymer precursors are utilized based on their suitability and availability for various applications. Among these, vegetable oil is one of the promising alternative bioresources for biomaterial synthesis due to its sustainability and availability in nature.

The potential of various tree parts from *Jatropha curcas* to efficiently treat wastewater effluents has been well documented in a few works. For example, biocoagulant extracted from *J. curcas* press cakes and seeds was found to be effective in reducing turbidity in kaolin wastewater [35], palm oil mill effluent (POME) [36], and pharmaceutical wastewater [37]. *J. curcas* bark, seed peel, and endosperm seed also showed excellent separation performance to remove divalent cadmium ions from wastewater [38]. Additionally, *Jatropha curcas* seed (chaff) adsorbent has successfully removed dye (Congo red) through the chemisorption process [39]. Our recent works synthesized *Jatropha* polyurethane membrane (JPU) mixed with 0.50 wt% graphene oxide (JPU/GO 0.50 wt%) and successfully removed Cu(II) ions from an aqueous solution. The JPU/GO 0.50 wt% membrane exhibits a dense defect-free surface morphology that contains active oxygenated functional groups due to the presence of graphene oxide (GO) [40].

Preliminary results showed that the JPU/GO 0.50 wt% membrane was able to provide a 71% rejection of Cu(II) ions at 100 ppm of feed concentration, 1.5 bar feed pressure, and pH 5. Therefore, it is important to access parameters that influence retention solutes for membrane performance optimization. For instance, feed concentration can have a substantial influence on osmotic pressure and hence, membrane performance. Apart from that, feed pressure can also be a significant factor. Upon application of pressure, solutes are suspended near the membrane surface, while water is forced to flow through the semi-permeable membrane. In a pressure-driven membrane system, concentration polarization or accumulation of solutes occur as the pressure increases, leading to a decline in the separation efficiency [41,42].

In addition, the pH of the solution also plays an important role in the filtration process, since pH affects membrane surface charge due to dissociation of the membrane functional groups [43]. In general, solution ionic strength (represented by the HCL concentration) is directly related to the Debye length (1/𝜅) of the membrane surface (or precisely, the membrane functional groups), which in turn, has a specific role in the formation of the double layer overlap that governs the electrostatic interaction for the separation process [44,45]. In other words, one can visualize the Debye length as an extension that reduces the effective size of the membrane pore [44]. By considering the presence of the functional groups in the membrane surface, the coupling effect of the Debye length (double layer overlap), which depends on membrane pore size, may become significant with a smaller pore diameter, and vice versa. With the right proportions, such variation may be a determining factor in the retention of a charged solute by a charged membrane [44].

All the above-mentioned factors can either independently or interrelatedly affect the Cu(II) ions removal. Thus, an optimum condition for the filtration process is essential to achieve higher Cu(II) ions rejection while maintaining high water flux. It is worth noting that to experimentally determine the effects of the above factors and interactions between each factor on Cu(II) removals may require hundreds of individual experiments, to a point where it is not practical. Hence, response surface methodology (RSM) software was used to design the filtration experiment for Cu(II) ions removal using a central composite model (CCD) [46]. RSM is a practical and commonly used method to monitor interactions between variables and to predict the overall effect of the parameters on the response (i.e., Cu(II) rejections). From RSM, an optimum condition for JPU/GO 0.50 wt% membrane to achieve the highest Cu(II) ions rejections can be determined. The input factors were feed concentration (ppm), pressure (bar), and solution pH. Prior to this, a parametric study, or screening process, was conducted by a single factor experiment, or one factor at a time (OFAT). This is important to establish the appropriate range of values to investigate the impact of each factor on the Cu(II) ions removal. Later, these experimental ranges for all factors were used as the feeder data for optimization purposes.

## 2. Materials and Methods

### 2.1. Materials and Reagents

Crude *Jatropha* oil (CJO) was supplied by Bionas Sdn Bhd, Kuala Lumpur, Malaysia. Glacial acetic acid was obtained from Fisher Scientific, Selangor, Malaysia while Amberlite IR-120, graphene oxide (GO), hexamethylene diisocyanate (HDI), and 30% hydrogen peroxide (H_2_O_2_) were supplied from Sigma-Aldrich, Selangor, Malaysia. Sodium carbonate (Na_2_CO_3_), copper sulfate (CuSO_4_∙5H_2_O), and methanol were obtained from R&M Chemicals, Selangor, Malaysia. Lastly, anhydrous sodium sulfate (Na_2_SO_4_) was attained from PC Laboratory Reagent, Selangor, Malaysia. All chemicals were used as received, without further purification. The experimental details for the preparation of JPU/GO 0.50 wt% for filtration can be found in our previous work [40].

### 2.2. Filtration Method

**Figure 1:**
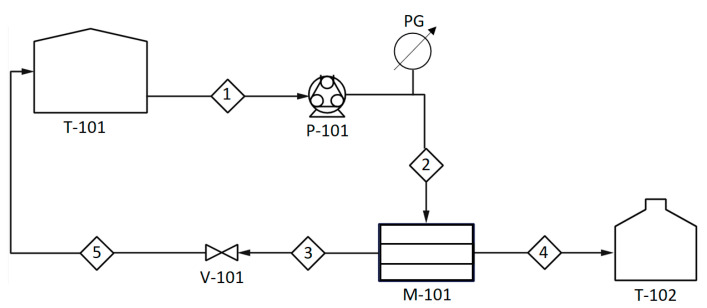
A schematic diagram of the experimental setup. Major stream, process unit, and instrumentations: feed tank (T-101), peristaltic pump (P-101), pressure gauge (PG), membrane module (M-101), control valve (V-101), retentate stream (3), and permeate stream (4). Modified based on our previous work [40].

A filtration experiment was conducted using a customized crossflow filtration system (Figure 1), and the details can be found in our previous work [40]. The concentration of Cu(II) ions was analyzed using atomic adsorption spectroscopy (AAS) and further used in Equation (1) to calculate the observed Cu(II) ions removal [41,42]:

(1) $$R_{o}=1-\frac{C_{p}}{C_{f}}$$

where $C_{p}$ and $C_{f}$ are the Cu(II) ions concentration in permeate and feed flow, respectively.

### 2.3. Determination of Zeta Potential at Different Solution pH Value

Zeta potential was analyzed using the Laser Particle Size Analyzer (LPSA) from Horiba, LA-960V2 model. The solution pH was adjusted from pH 2 to 5 using HCL (1.0 mol/L). The co-function of the double-layer overlap within the membrane pore size was studied to explore the mechanism of Cu(II) ions rejection. Here, the degree of the double-layer projection of the pore wall relies on the ionic strength of the electrolyte, and was described using the classical Debye–Huckel theory [47]:

(2) $$\kappa =\sqrt{\frac{2\times 10^{3}F^{2}I}{\varepsilon \varepsilon_{0}RT}}$$

where *F* is Faraday’s constant (96,485 C/mol), ε is the relative dielectric constant of a water molecule at 25 °C (78.6, dimensionless), $\varepsilon_{0}$ is the permittivity of the free space (8.85 × 10^−12^ F/m), *R* is the ideal gas constant (8.314 J/mol K), and *I* is the total ionic strength of the solution given by $I=\sum Cz^{2}$ (*C* is the molar concentration of the strong electrolyte and *z* is the number of charges). The inverse of κ defines the characteristic thickness (Debye length) of the double layer.

Based on Boltzmann statistics, the double-layer potential (φ) across the membrane pore as moving away from the pore wall towards the pore center can be predicted by the classical Gouy–Chapman model [45,48,49]:

(3) $$\varphi =\varphi_{0}exp\left(-\kappa d\right)$$

$\varphi_{0}$ (mV) is the surface potential of the membrane (ζ) [46], and *d* (nm) is the distance.

**Figure 2:**
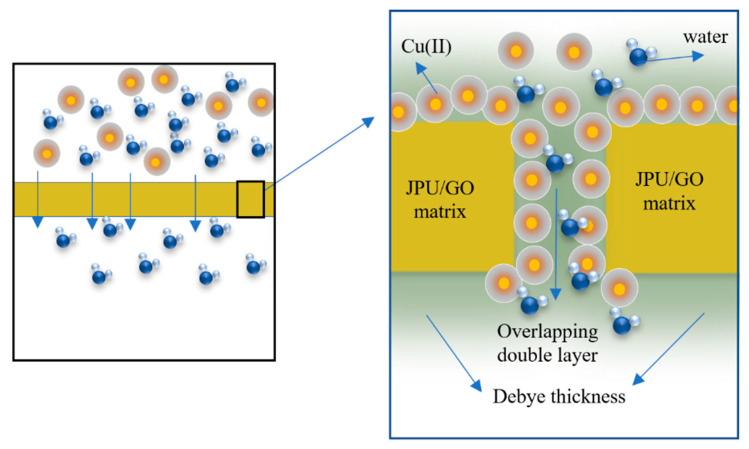
Proposed mechanisms for rejection of Cu(II) ions based on the charged exclusion effect within the pores JPU/GO 0.50 wt% membrane.

From Equation (3), double-layer potential (φ) is a function of surface chemistry and functional groups in the membrane matrix and properties of the electrolytes. Since the wall of the individual pore was assumed to be made of two face-to-face plates with double-layer potential coming into contact with each other, the overlapping potential was then added, as shown in Equation (4) [49]. Schematic illustrations of the double layer overlap in membrane separation mechanisms are presented in Figure 2.

(4) $$\sum \varphi =\varphi_{0}\left[exp\left(-\kappa d\right)+exp\left(-\kappa (d_{p}-d\right)\right]$$

($d_{p}$) is the pore diameter distribution and was proposed to be 10 Å from the previous report [50].

### 2.4. Parametric Studies by Single Factor Experiment

A sequential factor screening methodology, that is, a one factor at time (OFAT) design, was performed to determine the range of parameters. The selected variables were the initial feed concentration of Cu(II) ions, feed pressure, and solution pH, with the corresponding studied ranges of 60–160 ppm, 1.5–3 bar, and pH 2–5, respectively. The experiments were conducted by varying one factor at a time to investigate each factor’s effect on Cu(II) ions removal with their respective parameter ranges. Appropriate ranges for optimization studies were then confirmed.

### 2.5. Response Surface Methodology

Three independent variables, Cu(II) ions feed concentration, pressure, and pH of solutions, were the main factors considered for the rejection of heavy metals from an aqueous solution (Table 1). The experimental design was conducted using RSM to optimize the filtration condition and observe the interaction between factors affecting Cu(II) ions rejections. From the central composite design (CCD), 14 trial experiments were performed, with 3 repetitions at the center points.

The response was fitted by a second-order polynomial model, as shown in Equation (5) [51]:

(5) $$y=\beta_{0}+\overset{k}{\underset{i=1}{\sum }}\beta_{i}x_{i}+\overset{k}{\underset{i=1}{\sum }}\beta_{ii}x_{i}^{2}+\overset{k}{\underset{i=1}{\sum }}\overset{k-1}{\underset{j=i+1}{\sum }}\beta_{ij}x_{i}x_{j}$$

where *y* is the response variable (Cu(II) ions removal); *x_i_* and *x_j_* are the independent variables that influence the response *y*; *β_0_*, *β_i_*, *β_ii_*, and *β_ij_* are the coefficient for intercept, linear, quadratic, and interaction, respectively; and *k* is the number of variables.

## 3. Results and Discussion

### 3.1. Parametric Studies by Single-Factor Experiments

Initially, screening of the range of values to be used for subsequent RSM analysis was performed for feed concentration, pressure, and solution pH. The range of values studied were as follows; (a) feed concentration (60 ppm, 100 ppm, 140 ppm, 160 ppm); (b) water pressure (1.5 bar, 2.0 bar, 2.5 bar, 3.0 bar); and (c) pH value (2, 3, 4, 5).

#### 3.1.1. Effect of Cu(II) ions Feed Concentration

**Figure 3:**
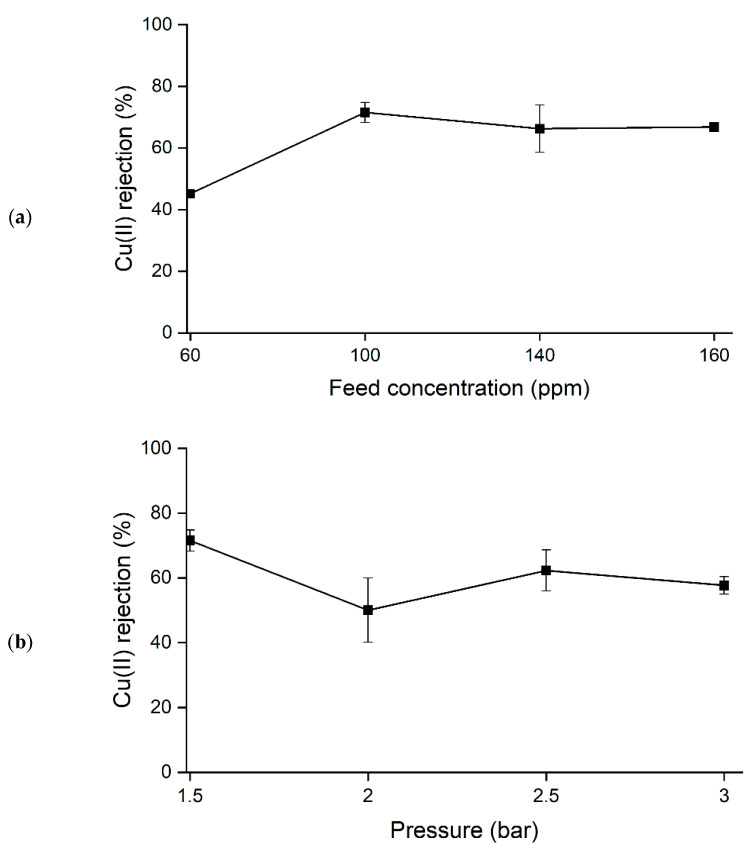

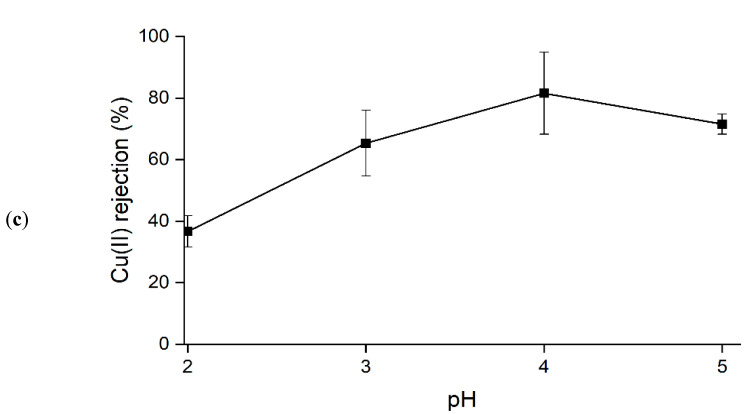
Cu(II) ions rejection (%) at different (**a**) feed concentration (ppm), (**b**) feed pressure (bar), and (**c**) pH value, respectively.

**Figure 4:**
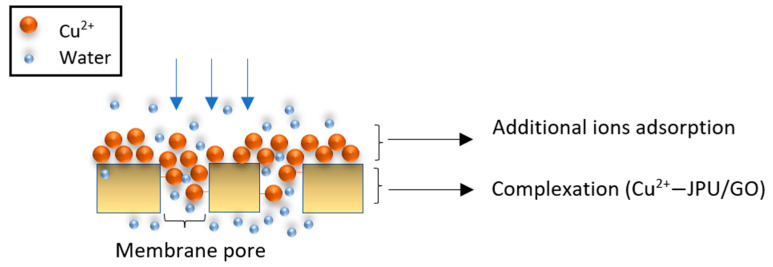
Schematic representation of JPU/GO 0.50 wt% membrane active layer during filtration.

The rejection of Cu(II) ions started to increase with the increase in feed concentration from 60 ppm to 100 ppm at constant pressure (1.5 bar) and pH value (pH 5), as shown in Figure 3a, and these conditions might be due to the changes in the membrane density, as reported by other researchers [52]. To date, it is believed that the membrane charge density is influenced by the bulk ion concentrations, in which membrane charge density increases with the electrolyte concentrations [53,54,55,56]. This phenomenon was caused by the selective and additional ions adsorption on the membrane surface or in the pore wall. Here, the first stage involved the complexation formation, followed by the adsorption of the additional ions, thus influencing the membrane charge [55,57,58]. Such behavior is schematically illustrated in Figure 4. This condition was possible due to the higher adsorption of the co-ions at the membrane interface, which increases the membrane charge density [52,57,59].

The increment of Donnan potential of the membrane with the membrane charge resulted in higher Cu(II) ions retention [52,57,59]. However, as the concentrations continued to increase (from 140 to 160 ppm), the Cu(II) ions rejection decreased to 66%. In this situation, excess feed concentration may cause more solutes to be transferred to the surface of the membrane and concentration polarization to be developed that eventually decreases the permeate flux due to the higher osmotic pressure [60,61]. Thus, the selected range parameters in feed concentration for the optimization in RSM were suggested to be from 60 to 140 ppm.

#### 3.1.2. Effect of Feed Pressure

The retentions of Cu(II) ions from the aqueous solution decreased with the increasing water pressure, from 71% to 57% rejection at constant feed concentration (100 ppm) and a pH 5 of the solution, as in Figure 3b. Commonly, higher water pressure tends to cause better membrane performance, as it pushes out more water while rejecting the solutes. Still, not all membranes follow the same behavior [62]. Higher water pressure is inclined to carry a higher amount of solutes on the membrane surface, causing concentration polarization, resulting in lower Cu(II) ions rejections. Emamjomeh et al. (2019) also found that higher water pressure caused a lower rejection of metal ions due to the concentration polarization of the solute on the surface membrane. Therefore, the feed pressure values for the optimization study in RSM were selected from 1.5 to 2.5 bar.

#### 3.1.3. Effect of Solution pH Value

The rejection of Cu(II) ions at lower pH decreased with constant feed pressure (1.5 bar) and feed concentration (100 ppm), as shown in Figure 3c. To study the effect of pH on the filtration process, a few mechanisms must be considered. According to the classical transport models, the membrane’s selectivity and permeability are mainly based on two mechanisms, namely the sieving effect or the charge effect, depending on the properties of the membrane and the solute [63,64]. The sieving effect can dominate the rejection process only when the pore size of the membrane is smaller than the solute; otherwise, solute penetration may occur. On the contrary, the surface charge effect, based on the electrostatic interaction, can be the determining factor when the membrane pores are larger than the solute. This charged state can be measured using the zeta potential [65,66,67]. In elucidating the membrane mechanisms, the zeta potential measurement for JPU/GO 0.50 wt% membrane in distilled water and Cu(II) ions solution, with the calculation on the double-layer overlap within JPU/GO 0.50 wt% pore membrane at different pH, is essential. In this experiment, filtration of Cu(II) ions from the JPU/GO 0.5 wt% membrane was conducted at different pH by using HCl to alter the pH value of the electrolyte.

**Figure 5:**
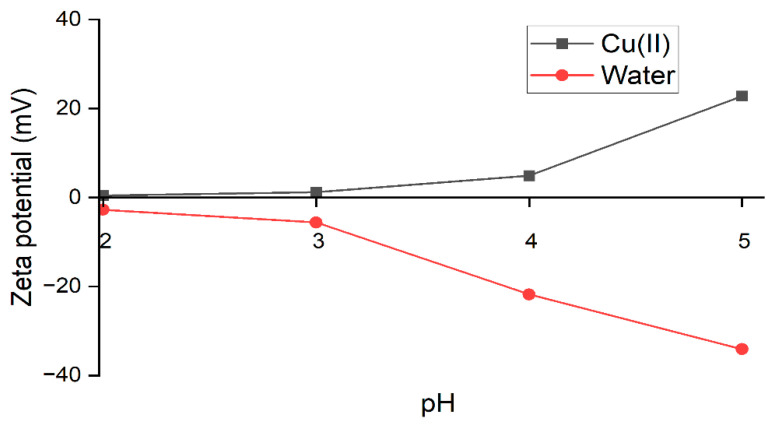
Zeta potential (mV) on JPU/GO 0.50 wt% membrane at different pH in distilled water and in Cu(II) ions solution.

As shown in Figure 5, it was found that JPU/GO 0.50 wt% membrane in distilled water possessed a negative zeta potential value at −34.1, −21.8, −5.6, and −2.7 mV for pH 5, 4, 3, and 2, respectively. Studies have found that pH affects the charge of a membrane due to the disassociation of the functional groups [68]. The negative values of the membrane charge were decreasing towards lower pH values, due to the higher protons concentration that caused the protonation of the hydrophilic functional groups [69,70].

On top of measuring the zeta potential of the membrane in pure water, we also measured the zeta potential of the JPU/GO 0.50 wt% membrane in Cu(II) ions solution to elucidate the change in membrane charge during filtration that may potentially affect its filtration mechanism [45]. From Figure 5, the zeta potential of the membrane was obtained as 22.8, 4.9, 1.2, and 0.4 mV for pH 5, 4, 3, and 2, respectively. At normal pH 5 (±0.5), the membrane charge was observed to increase drastically, and this might be attributed to the adsorption of Cu(II) ions on the membrane surface [71,72]. Since the membrane charge was originally negative, the cation (Cu(II) ions) is believed to play a more dominant role in surface charge acquisition and thus, a higher amount of Cu(II) ions were readily adsorbed onto the membrane surface, which shifted the zeta potential to positive 22 mV [71,72]. However, at lower pH (2,3, and 4), due to the presence of both Cu(II) ions and HCl, the close approach of the co-ions (${\mathrm{Cl}}^{-}$) appeared to counteract some of the effects of the adsorbed Cu(II) ions onto the membrane surface, causing only a slight change in membrane charge from the original charge value [71].

Based on the results, higher Cu(II) ions rejection was obtained at pH 3 to 5. This condition might be due to the shifted positive membrane charge that favored the rejection of Cu(II) ions under repulsion by Donnan exclusion [54,69]. On the other hand, at pH 2, Cu(II) ions rejection drastically reduced to 37% due to the weak surface charge of the membrane as it approached the isoelectric point IEP (the pH at which the membrane has a zeta potential with zero charge) [73]. The closer the absolute value of the zeta potential to the IEP, the higher the possibility for the membrane to reach the critical point of zeta potential at which the surface charge effect became less effective [69].

In addition, it is reasonable to consider the presence of the oxygenated functional groups in the membrane polymer matrix [45]. The distance between two functional groups in membrane pores was assumed to develop the double-layer overlap potential (φ) that aided in the Cu(II) ions rejection [45]. Beforehand, the double-layer overlap potential (φ) within the membrane pore size was in co-function with the degree of the electrostatic double layer, (Debye length) of the pore wall. Using the classical Debye–Huckel theory [46], from Equation (2), the Debye length (1/κ*)* of the membrane pore was estimated to be at 3.8, 3.7, 2.9, and 1.6 nm corresponding to pH 5, 4, 3, and 2, respectively. After obtaining the Debye length, it is now possible to estimate the double-layer overlap potential that affected the retention of Cu(II) ions using Equation (4).

**Figure 6:**
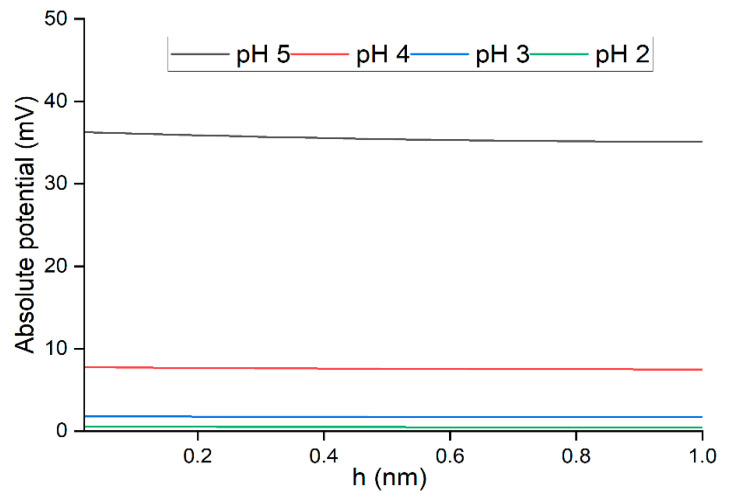
Distribution of double-layer overlap potential (mV) of JPU/GO 0.50 wt% membrane at different pH.

Based on the results in Figure 6, at a lower pH value, the reduction in the double-layer overlap was observed at lower pH due to the double-layer compression at higher ionic strength due to the presence of HCl for regulating the pH [45]. With the significant reduction in the overlap potential within the membrane interfaces, the membrane selectivity had become less effective, thus lowering the metal ion rejection at pH 2. Taken together, it can be concluded that the rejection of Cu(II) ions at pH 2 decreased due to the interrelated factors, such as the weakling of the membrane surface charge and the disappearance of the overlapping potential at pH 2 due to the higher ionic strength. Thus, for the optimization in RSM, pH values from 3 to 5 were selected for higher Cu(II) ions rejection.

### 3.2. Optimization of Cu(II) ions Filtration by Response Surface Methodology (RSM)

The input factors are the feed concentration (A), pressure (B), and solution pH value (C). The experiment was conducted based on the experimental design, and the results are shown in Table 2.

#### 3.2.1. Model Fitting

The responses from the experimental data of Cu(II) ions rejection were fitted into these four models: linear, 2FI (two factorial), quadratic, and cubic models to determine the regression equation. The statistical significance of the models was evaluated using the *F*-test and *p*-value from the analysis of variance (ANOVA). Based on Table 3, two models were suggested: quadratic and linear, while the cubic model was aliased.

**Figure 7:**
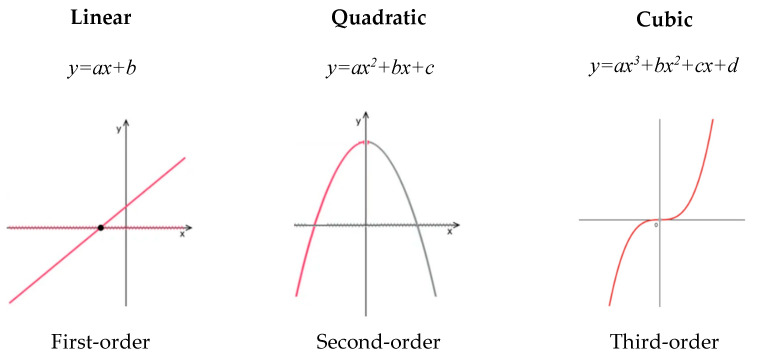
Polynomial shapes with their functions [46].

In general, a linear model is a linear polynomial that depicts the results in linear form; a quadratic model is a second-degree non-linear polynomial model that describes the results in a curvature graph, and the cubic model is a third-degree non-linear polynomial model that describes the results from the cubic equation (Figure 7). In addition, the quadratic model also involves interaction between all factors that influence the response. Commonly, models in RSM are selected based on the highest order polynomial while at the same time, the model is not aliased [46,74]. Thus, based on the fundamental assumption mentioned earlier, the quadratic model was selected due to its higher degree of polynomial rather than linear model.

#### 3.2.2. Analysis of Variance (ANOVA)

To understand the significance of the fitted model, an analysis of variance (ANOVA) was conducted. Based on Table 4, the predicted model’s R-squared (R^2^) value was 0.9877, implying that the model exhibited a good fit for the experimental data [75]. Thus, this value revealed that 98.77% of the variation in the responses (Cu(II) ions rejection) were well described by the effect of the individual model terms involved (filtration factors). However, Halim et al. (2021) reported that R^2^ continued to increase with more added terms, without considering the significance of each model term. Thus, the adjusted-R^2^ (adj-R^2^) and predicted-R^2^ (pred-R^2^) are more reliable in analyzing the model fitting [41]. Both adj-R^2^ and pred-R^2^ values depend on the significant terms considered in any work [76]. For example, as more significant terms are added, the higher the value of adj-R^2^, which denotes more significant terms in the model. In contrast, the value of adj-R^2^ will decrease when the non-significant terms are involved in predicting the model [76]. Meanwhile, pred-R^2^ represents the ability of the model to predict the new observation, depending on the model’s significant terms [51].

From the explanation above, the obtained adj-R^2^ value was 0.9601 (96.91%) which signified the model’s accuracy in describing the relationship between the response and factors [51]. However, the obtained pred-R^2^ value was −0.1643 (−16.43%), indicating that the model was overfitting [77]. An overfit model is a model that contains too many predictors, which causes it to start modeling the random noise [77,78]. Since predicting the random noise was impossible, the pred-R^2^ value was dropped for an overfit model, thus demonstrating unfit data [77,78]. From the findings, it was revealed that the model contained insignificant terms, which were examined based on the *p*-value. Based on the data obtained, variables AC, BC, and B^2^ exhibited higher *p*-value (>0.1), thus resulting in a negative Pred-R^2^.

Apart from that, the model’s validity was also evaluated based on other statistical properties, such as adequate precision, coefficient of variation (CV%), and residual error sum of squares (PRESS). Adequate precision evaluates the signal-to-noise ratio, which assesses the range of the predicted values at the design points to the average prediction variance [51]. A value higher than 4 indicates that the model can differentiate the source of data variations. The obtained adequate precision value from this model was 19.358, suggesting that the model was adequate to remove the unnecessary design points based on the parameters’ interest [79]. Moreover, the CV% value indicates the dispersion degree of the data points around the mean value; a smaller value (<10%) denotes a good reproducibility of the model [80,81]. From this model, the obtained CV% was 6.34%, indicating the model’s reproducibility. Finally, the PRESS value describes the model’s capability to fit each point in the design by the fitting model to all design points, except the predicted one [51]. The residuals values are then squared and summed to give the PRESS value, as reported in ANOVA. A smaller value is desirable, which signifies a high value of Pred-R^2^. However, the obtained PRESS value from this model was considerably large (6508.51), which was interrelated with the low value of Pred-R^2^.

Based on the results obtained above and to improve the accuracy of the predicted model, further work was conducted to remove a statistically non-significant term from the analysis using a reduction method [78]. The reduction was made step-by-step using backward elimination (from the most insignificant term) until a higher pred-R^2^ value was achieved [78,82]. From this analysis, the elimination terms with a *p*-value larger than 0.5 were found to exhibit the best prediction for Cu(II) ions rejection. From the reduction method, a higher pred-R^2^ value was obtained at 0.8878 (88.78%), suggesting that the reduced model had a good predictive ability. Furthermore, the newly obtained R^2^ and adj-R^2^ values were constantly high at 0.9875 (98.75%) and 0.9676 (96.76%), respectively. Meanwhile, the reduced model also increased the value of lack of fit (LOF) and adequate precision to 0.4970 and 22.458, respectively. Moreover, the obtained CV% and PRESS values were reduced to 5.71% and 627.23, respectively. Therefore, from the analysis, the model’s precision and its predictive ability were improved. The final empirical models, after excluding the insignificant term (BC), and (*Y*) were mentioned in Equation (6). From the equation, it can be concluded that it involved both the linear effects and the interaction between each parameter for predicting the Cu(II) ions rejection.

(6) $$\left(Y\right)=81.11-4.28\;A-5.48\;B-10.13\;C-19.73\;AB+2.86\;AC-14.52\;A^{2}-4.84\;B^{2}-10.49\;C^{2}$$

where *A* = concentration of feed (ppm), *B* = pressure (bar), and *C* = solution pH value. *A* positive sign denotes the synergistic impact, while a negative sign indicates the resistivity effect, based on the factors in the equation [83].

#### 3.2.3. Residual Analysis

**Figure 8:**
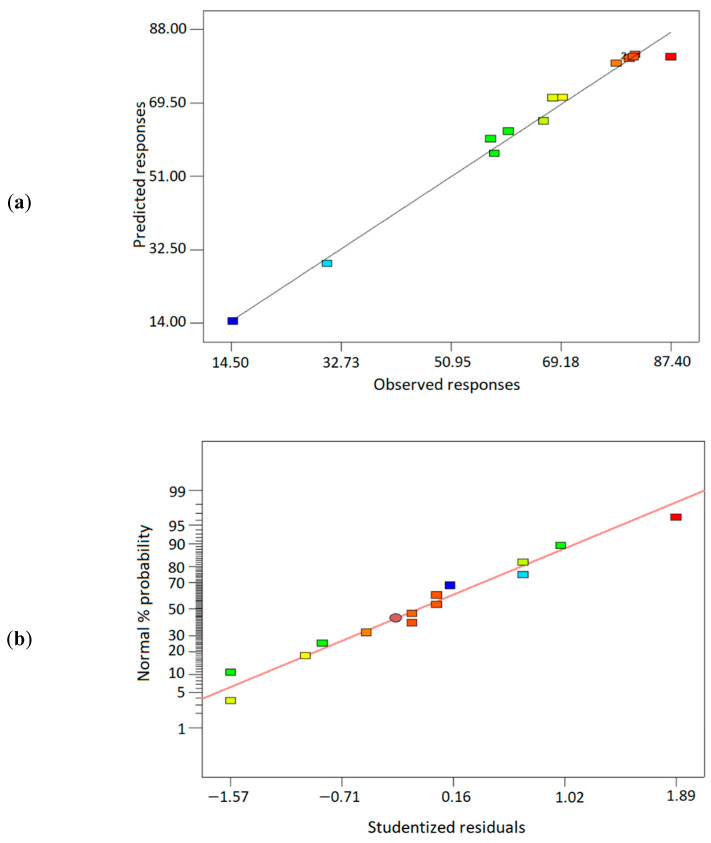

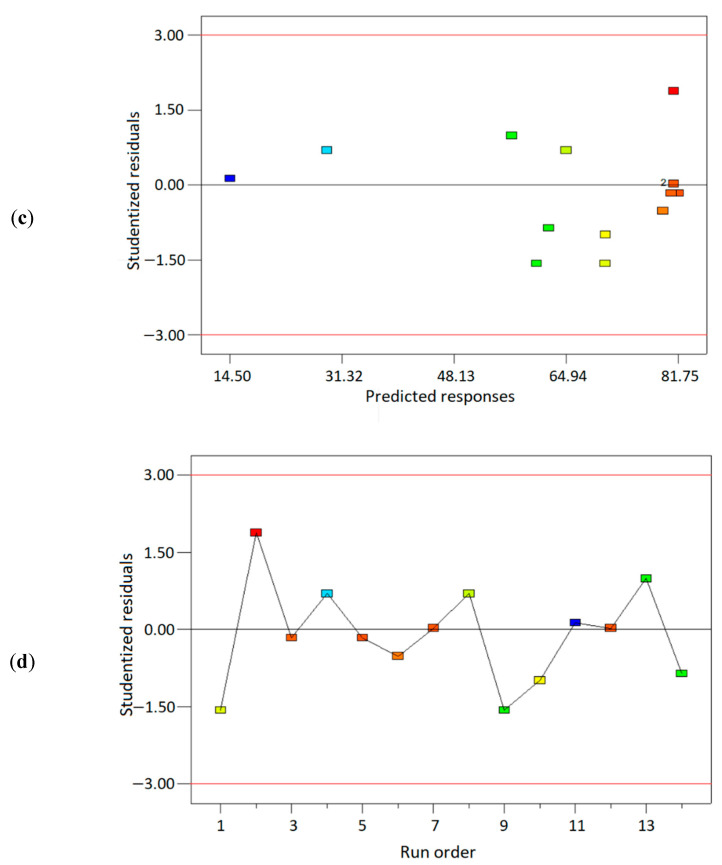
The residuals analysis diagnostic plots for (**a**) predicted versus observed responses, (**b**) normal probability plot, (**c**) residuals versus predicted responses, and (**d**) residuals versus run order.

Residual analysis is important to assess the residual distribution and identify the outliers [84]. Thus, four diagnostic plots were analyzed, as shown in Figure 8: (a) predicted responses vs. observed responses, (b) normal probability plot, (c) residuals vs. predicted responses, and (d) residuals vs. run order. From Figure 8a, the actual versus predicted graph shows that the model and each response’s prediction did not deviate far, following the normal line. Similarly, based on the normal probability plot in Figure 8b, the scattered residuals values were distributed along the normal line, indicating the ANOVA’s adequacy [84]. Moreover, as shown in Figure 8c, the homogeneity of the variances was analyzed based on the residuals plots versus the predicted response. The residuals points were scattered randomly on both sides of the normal line, which signified the consistency of the variances [51]. Lastly, the residuals plots versus run order, as depicted in Figure 8d, revealed that the residuals points were distributed around the normal line without any patterns, which indicated that the residuals are independent of each other, thus obeying the ANOVA assumptions. Additionally, the scattered residual points from diagnostic plots in Figure 8c,d exhibited no outliers, denoting that the residual points were only distributed randomly within the upper and lower limit lines [85]. Thus, based on the residuals analysis, ANOVA assumptions were fulfilled, and it can be concluded that the regression model yielded unbiased coefficient estimations with the minimum variance.

#### 3.2.4. Effect of Filtration Factors Based on the Response Surface Plots Analysis

**Figure 9:**
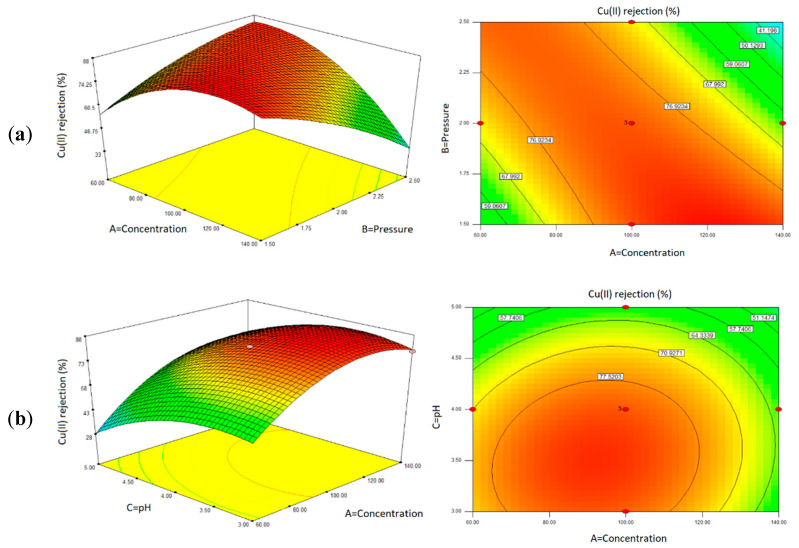
Response surface plots of the interaction effects of filtration factors on the Cu(II) ions rejection: (**a**) concentration and pressure (AB) and (**b**) concentration and pH (AC).

The interaction effects between the independent factors and the responses were illustrated using response surface plots in two-dimensional (2D) contour plots and three-dimensional (3D) surface plots, as depicted in Figure 9. From the polynomial model in Equation (6), the 3D surface plots were created by considering the correlation effects of two factors at a time, while the other factor was held constant at middle-level values. The variation of colors from the plots, such as red, green, and blue, indicates the maximum, medium, and minimum level of Cu(II) ions rejection, respectively.

Figure 9a presents a response surface plot of the reduced quadratic model with a function of concentration (A) and pressure (B) at a constant pH of 3.7. Based on the plot, it could be observed that the maximum rejection of Cu(II) ions was achieved at the optimum feed concentration (100 ppm) and lower feed pressure (1.5 bar). Higher feed concentration would influence the membrane charge density, in which more adsorption of additional co-ions onto the membrane surface increased the Donnan potential, thus promoting higher Cu(II) ions from Donnan exclusion [52,57,59]. However, at elevated feed concentration (140 ppm) with higher feed pressure (2.0–3.0 bar), Cu(II) ions rejection was decreased due to the higher rate of solute transported towards the membrane surface, which caused excessive solute accumulations, thus resulting in the concentration polarization [62].

Moreover, Figure 9b, a response surface plot with a function of pH (C) and feed concentration (A) at constant 1.5 bar pressure, revealed that higher Cu(II) ions rejection was achieved at the optimum concentration (100 ppm), with a lower pH value range from pH 3 to 4. The filtration at a lower pH (3 and 4) contained the additional HCl as the background electrolyte. The close approach of the co-ions from ${\mathrm{SO}}_{4}^{2-}{\mathrm{and}\;\mathrm{Cl}}^{-}$ ions reduced the adsorption of cation, Cu(II) ions on the membrane surface, which reduced the membrane resistance, resulting in the negligible change of the membrane performance [71].

#### 3.2.5. Optimization and Model Validation

The optimization aims to maximize the Cu(II) ions rejection at the suggested conditions. Based on Table 5, the optimum condition was proposed at 116 ppm, 1.5 bar, and at pH 3.7 to obtain the maximum Cu(II) ions rejection (87%), based on the desirability function of 1, which indicates the reasonable approximation of the prediction point. Subsequently, an experiment was carried out based on the suggested optimum operating conditions and achieved 82% of Cu(II) ions rejection, with a 6% error, as reported in Table 6. Moreover, Cu(II) ions rejection fell within the 95% prediction intervals, which signified the accuracy of the regression model.

## 4. Conclusions

In conclusion, a regression model was generated based on the experimental design. Optimization was performed based on the model design, producing 82% of Cu(II) ions removal with the optimum conditions at 116 ppm Cu(II) ions feed concentration, 1.5 bar, and solution pH 3.7. From the optimization, feed pressure, and solution pH value were observed to be more significant in the lower range, while a higher feed concentration was found to produce higher Cu(II) ions removal. However, it was apparent that too high a feed concentration resulted in a gradual decrease in rejection, due to a concentration polarization condition [60,61,62]. Additionally, the reduction model offered a better estimation for Cu(II) ions removal with the elimination of insignificant model terms with a *p*-value larger than 0.5. The quadratic regression model was found to be significant (R^2^ = 0.98) for the optimization prediction, with the experimental result in the 95% predicted interval ranges (95% low/high).

## Figures and Tables

**Table 1:** Experimental design summary in RSM for Cu(II) ions removal by using 0.50 wt% JPU/GO membrane.

Symbol	Factor	Low Level	Middle	High Level
A	Concentration (ppm)	60	100	140
B	Pressure (bar)	1.5	2	2.5
C	pH value	3	4	5

**Table 2:** RSM data response for Cu(II) ions removal from an aqueous solution.

No.	Factor A: Concentration (ppm)	Factor B: Pressure (bar)	Factor C: pH	Response 1: Cu(II) Ions Rejection (%)
1	60	2.0	4	70
2	60	2.5	5	58
3	100	1.5	4	82
4	100	2.0	3	80
5	100	2.0	4	87
6	100	2.0	4	81
7	100	2.0	4	81
8	100	2.0	5	58
9	100	2.5	4	68
10	140	1.5	3	78
11	140	1.5	5	66
12	140	2.0	4	60
13	140	2.5	3	30
14	140	2.5	5	15

**Table 3:** Sum of squares in a subsequent model.

Source	Sum of Squares	Df	Mean Square	*F*-Value	*p*-Value Prob > *F*	Remarks
Mean	59,800.48	1	59,800.48	-	-	-
Linear	4054.09	3	1351.36	8.80	0.0037	Suggested
2FI	370.89	3	123.63	0.74	0.5596	-
Quadratic	1096.34	3	365.45	21.32	0.0064	Suggested
Cubic	42.94	2	21.47	1.68	0.3737	Aliased
Residual	25.63	2	12.81	-	-	-
Total	65,390.37	14	4670.74	-	-	-

**Table 4:** Analysis of variance (ANOVA) of the quadratic model.

Source	Sum of Squares	Df	Mean Square	*F*-Value	*p*-Value
Model	55.27	9	613.48	35.79	0.0018
A (Concentration)	79.56	1	55.27	3.22	0.1470
B (Pressure)	275.13	1	79.56	4.64	0.0975
C (Ph)	722.57	1	275.13	16.05	0.0160
AB	14.14	1	722.57	42.15	0.0029
AC	1.16	1	14.14	0.82	0.4152
BC	486.37	1	1.16	0.068	0.8075
A^2^	53.68	1	486.37	28.37	0.0060
B^2^	253.67	1	53.68	3.13	0.1515
C^2^	55.27	1	253.67	14.80	0.0184
Residual	68.57	4	17.14		
Lack of fit (LOF)	42.94	2	21.47	1.68	0.3737
Pure error	25.63	2	12.81		
Corrected total	5589.89	13			
Statistical analysis of the regression equation					
R^2^	0.9877	Standard deviation (SD)			4.14
Adjusted-R^2^	0.9601	Mean			65.36
Predicted-R^2^	−0.1643	Coefficient of variation (CV%)			6.34
Adequate precision	19.358	PRESS			6508.51

**Table 5:** Numerical optimization and model validation for JPU/GO 0.50 wt%.

Factor/ Response	Level	Optimization Goal	Lower Limit	Upper Limit	Prediction Point
A	Concentration (ppm)	In range	60	140	116
B	Pressure (bar)	In range	1.5	2.5	1.5
C	Acidity (pH)	In range	3	5	3.7
Response Y	Cu(II) ions Rejection (%)	Maximize	14	87	87

**Table 6:** Model validation.

Predicted Response	Desirability	95% PI Low	95% PI High	Observed Response	Error (%)
87	1	76	99	82	6

## Data Availability

For this section, the supporting data is based on submitted doctoral dissertation thesis, which is not yet being published/available online. Thus, available upon request.
